# FPGA-Based Implementation of Multidimensional Reconciliation Encoding in Quantum Key Distribution

**DOI:** 10.3390/e25010080

**Published:** 2022-12-31

**Authors:** Qing Lu, Zhenguo Lu, Hongzhao Yang, Shenshen Yang, Yongmin Li

**Affiliations:** 1 State Key Laboratory of Quantum Optics and Quantum Optics Devices, Institute of Opto-Electronics, Shanxi University, Taiyuan 030006, China; 2 Collaborative Innovation Center of Extreme Optics, Shanxi University, Taiyuan 030006, China; 3 College of Physics and Information Engineering, Shanxi Normal University, Taiyuan 030031, China; 4 Key Laboratory of Spectral Measurement and Analysis of Shanxi Province, Shanxi Normal University, Taiyuan 030031, China

**Keywords:** continuous variable quantum key distribution, multidimensional reconciliation, field-programmable gate array, encoding, variable throughput

## Abstract

We propose a multidimensional reconciliation encoding algorithm based on a field-programmable gate array (FPGA) with variable data throughput that enables quantum key distribution (QKD) systems to be adapted to different throughput requirements. Using the circulatory structure, data flow in the most complex pipeline operation in the same time interval, which enables the structural multiplexing of the algorithm. We handle the calculation and storage of eight-dimensional matrices cleverly to conserve resources and increase data processing speed. In order to obtain the syndrome more efficiently, we designed a simplified algorithm according to the characteristics of the FPGA and parity-check matrix, which omits the unnecessary operation of matrix multiplication. The simplified algorithm could adapt to different rates. We validated the feasibility and high speed of the algorithm by implementing the multidimensional reconciliation encoding algorithm on a Xilinx Virtex-7 FPGA. Our simulation results show that the maximum throughput could reach 4.88 M symbols/s.

## 1. Introduction

Quantum cryptography can provide information-theoretic security by combining one-time pad with quantum key distribution (QKD) [1,2] and has become an important branch and hotspot in the field of modern cryptography. QKD allows legitimate parties, Alice and Bob, to share secure keys even if the quantum channel is controlled by eavesdropper Eve. The fundamental theorems of quantum physics (no-cloning theorem, etc.) guarantee that quantum states transmitted through a quantum channel cannot be replicated accurately. Any eavesdropping behavior inevitably disturbs the quantum states on which the key information is encoded, which results in the increase in channel noises. Such noises can be monitored by the legitimate parties; therefore, any eavesdropping behavior can be discovered.

According to different carriers of the key, QKD can be divided into discrete-variable QKD (DVQKD) and continuous-variable QKD (CVQKD) [3,4,5,6]. DVQKD uses the polarization or phase degree of single photons to encode key information, which can realize long-distance key distribution using single-photon detection technology and post-selection. CVQKD employs the quadrature components of quantum states to encode key information; it is compatible with existing optical communication technology, and the key rate is high for short and medium distances. Important progress has been made recently [7,8,9,10,11,12,13,14]. However, the data post-processing of CVQKD is relatively complex because it usually works at a low-SNR regime.

A typical CVQKD system usually consists of three parts: (1) preparation, distribution, and measurement of quantum states; (2) data sifting and parameter estimation; (3) data post-processing process [15]. The last part can be divided into two stages, information reconciliation and privacy amplification. In the information reconciliation stage, Alice and Bob obtain the same binary keys by correcting the data errors between their raw keys. After information reconciliation, Alice and Bob extract the final secret key using privacy amplification techniques.

At present, there are two schemes for data reconciliation in the CVQKD system, slice reconciliation [16] and multidimensional reconciliation [17], which are suitable for different SNR ranges. Slice reconciliation is suitable for relatively high SNRs, i.e., larger than 1 (short transmission distance) [18,19], and multidimensional reconciliation is suitable for low SNRs, from 0.01 to 1 (long transmission distance) [13,20]. In the case of Gaussian-modulated CVQKD, the multidimensional reconciliation algorithm provides a powerful encoding scheme for low-SNR scenarios and thus effectively extends the key distribution distance. In this way, the channel between Alice and Bob is converted into a virtual binary input additive white Gaussian noise (AWGN) channel; therefore, efficient binary codes can be employed.

In quantum information processing, error-correcting codes play a very critical role in protecting information from noise interference [21]. In CVQKD, an error correction code (ECC) with a large block is required to obtain high reconciliation efficiency, which is crucial for system performance. Because multiple iterations and low-density parity-check (LDPC) codes [22] are required in data reconciliation, the required computation complexity is high. On the other hand, the repetition rate of the CVQKD system increases rapidly. In this case, the speed of the corresponding data post-processing should match it; otherwise, the actual key rate would be reduced. The throughput of error correction algorithms based on general central processing units (CPUs) is very limited. A feasible solution to increase throughput is to utilize hardware-based acceleration, such as Graphics Processing Units (GPUs) [23,24,25,26,27] and field-programmable gate arrays (FPGAs) [15,28], which dramatically improve the computation speed. FPGAs are very attractive when designing prototypes and are applicable to small-scale production. They have high processing speed, parallelism, re-programmability, and low power consumption. The last one provides them with good integration abilities.

Multidimensional reconciliation algorithms have not been realized on an FPGA platform. In this paper, we achieve the encoding algorithm of multidimensional reconciliation with variable data throughput on an FPGA. To this end, we use flexible division of pipeline operation combined with an equal-interval circulatory structure, which can achieve different throughput. The calculation and storage of eight-dimensional matrices is cleverly designed to conserve resources and increase data processing speed. The high data processing speed of encoding can increase the real-time secret key rate of the CV-QKD system. Furthermore, a simplified algorithm without operation of matrix multiplication is exploited according to the characteristics of the FPGA and parity-check matrix to construct the syndrome efficiently.

The rest of this paper is organized as follows: In Section 2, we present the principles and steps of multidimensional reconciliation. In Section 3, we present the design and the detailed implementation of the multidimensional algorithm on an FPGA chip. The performance of the algorithm is analyzed in Section 4. In Section 5, we give a summary.

## 2. The Principle of Multidimensional Reconciliation

**Figure 1:**
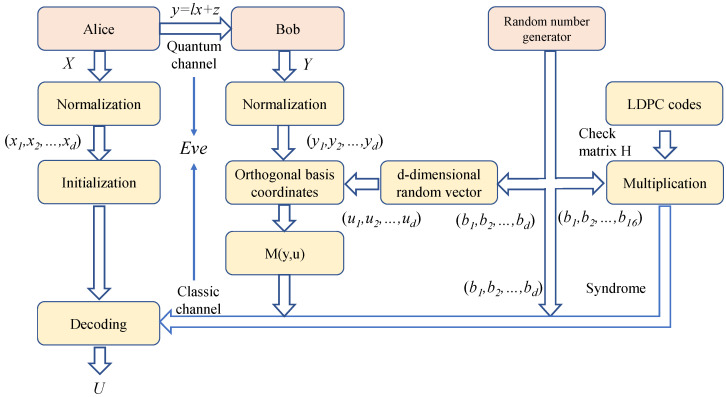
Diagram of the multidimensional reconciliation scheme. Here, d-dimensional random vector u is generated from a random binary sequence $\left(b_{1},b_{2},\ldots ,b_{8}\right)$. Random binary sequence $\left(b_{1},b_{2},\ldots ,b_{16}\right)$ and parity-check matrix *H* are multiplied to obtain the syndrome.

For Gaussian modulation CVQKD protocols, the shared raw keys of Alice and Bob are correlated Gaussian variables. For long transmission distances, the SNR is very low. In this case, the Gaussian variables have a small absolute value and are distributed around 0; thus, it is difficult to discriminate the sign and realize the encoding. The basic ideal of multidimensional reconciliation [17] is that one can perform a proper rotation before encoding the key in the sign of the coordinates; in this way, the small-absolute-value coordinates can be eliminated. The schematic diagram of multidimensional reconciliation is shown in Figure 1. The encoding ideal is as follows: Given $y\in S^{n-1}$ ($S^{n-1}$ represents a sphere), define a cube $Q_{y}$ centered on 0 and containing *y* such that the prior distribution of *y* in $Q_{y}$ is uniform. The description of $Q_{y}$ can be achieved by describing the orthogonal transformation of the transformed canonical cube. Bob randomly selects vertices *U* ($U\in S^{n-1}$) of the canonical cube and then provides Alice with an orthogonal transformation M(y,U) that maps *y* to *U*, satisfying M(y,U)y=U; this transformation defines cube $Q_{y}$. Finally, Alice can recover *U* according to *x* and M(y,U). The specific steps of multidimensional reconciliation are reported below.

Step 1. For security, Alice and Bob use the method of sequential combination to form a d-dimensional vector of *d* continuous variables in multidimensional reconciliation and obtain $X=(x_{1},x_{2},\ldots ,x_{d})$ and $Y=(y_{1},y_{2},\ldots ,y_{d})$, where *d* denotes the dimension of multidimensional reconciliation. Here, we choose d=8 because its performance with a low SNR is better than that of other dimensions (d=1,2,4) [29]. Assuming that the quantum channel is linear, the relationship between Alice and Bob can be expressed as

(1) $$\begin{array}{c} y=lx+z, \end{array}$$

and

(2) $$\begin{array}{c} x∼N(0,\sigma_{x}^{2}), \end{array}$$

(3) $$\begin{array}{c} z∼N(0,\sigma_{z}^{2}). \end{array}$$

where *x* and *y* represent the d-dimensional vectors of Alice and Bob, respectively; and *z* represents the noise of the quantum channel.

Step 2. In order to change the variable space from a non-uniform Gaussian distribution to a uniform Gaussian distribution, the d-dimensional state points need to be mapped from Euclidean space $D^{d}$ to unit spherical space $S^{d-1}$. To this end, Alice and Bob normalize their Gaussian variables *x* and *y*.

(4) $$\begin{array}{c} x=\frac{x}{∥x∥}, \end{array}$$

and

(5) $$\begin{array}{c} y=\frac{y}{∥y∥}, \end{array}$$

where

(6) $$\begin{array}{c} ∥x∥=\sqrt{\langle x,x\rangle }, \end{array}$$

and

(7) $$\begin{array}{c} ∥y∥=\sqrt{\langle y,y\rangle }. \end{array}$$

Step 3. Bob generates a random binary sequence $\left(b_{1},b_{2},\ldots ,b_{d}\right)$. Then, a d-dimensional random vector $u=(u_{1},u_{2},\ldots ,u_{d})$ can be generated as

(8) $$\begin{array}{c} u=[\frac{{\left(-1\right)}^{b_{1}}}{\sqrt{d}},\frac{{\left(-1\right)}^{b_{2}}}{\sqrt{d}},\ldots ,\frac{{\left(-1\right)}^{b_{d}}}{\sqrt{d}}]. \end{array}$$

Step 4. Bob calculates rotation matrix M(y,u). When d=8, there is a (non-unique) family of 8 eight-dimensional orthogonal matrices $\left(A_{1},A_{2},..,A_{d}\right)$ [17], where $A_{1}$ is an 8 × 8 identity matrix. Moreover, for i,j>1, we have

(9) $$\begin{array}{c} \left\{A_{i},A_{j}\right\}=-2\delta_{ij}A_{1}, \end{array}$$

where {A,B}=AB+BA, $\delta_{ij}$ is the unit impulse function. Rotation matrix M(y,u) can be calculated from a family of d-dimensional orthogonal matrices $\left(A_{1},A_{2},..,A_{d}\right)$ as

(10) $$\begin{array}{c} M\left(y,u\right)=\sum_{i=1}^{d}\alpha_{i}\left(y,u\right)A_{i}, \end{array}$$

where, $\alpha_{i}\left(y,u\right)=\left(A_{i}y|u\right)$ are the coordinates of *u* in basis $\left(A_{1}y,A_{2}y,\ldots ,A_{d}y\right)$ and $\left(A_{1}y,A_{2}y,\ldots ,A_{d}y\right)$ is a set of standard orthonormal bases for multidimensional space. The above proves that M(y,u)y=u.

Step 5. Bob calculates the syndrome using parity-check matrix *H* and random binary sequence $(b_{1},b_{2},\ldots ,b_{16}).$

(11) $$\begin{array}{c} I_{syn}=H\cdot \left(b_{1},b_{2},\ldots ,b_{16}\right), \end{array}$$

where $I_{syn}$ is the syndrome and $\left(b_{1},b_{2},\ldots ,b_{16}\right)$ is a 16-bit binary string. The width of the binary string is determined by the structure of LDPC codes.

## 3. FPGA Logic Design and Implementation of Multidimensional Reconciliation Encoding

In the FPGA design and implementation, we used the pipeline operation to achieve high throughput with minimal FPGA resources and theoretically analyzed the throughput of our scheme. Then, we designed the storage mode of an eight-dimensional matrix according to the addressing mode of the FPGA and simplified the calculations for the orthogonal basis coordinates and matrix inversion of the eight-dimensional matrix. Finally, the syndrome was obtained through addressing and shifting.

### 3.1. Hardware Implementation Scheme

In order to implement the multidimensional reconciliation algorithm on an FPGA chip, we used VC709 Evaluation Kit manufactured by Xilinx, which included a Virtex-7 VX690T FPGA [30] and a user-programmable differential oscillator (range: 10 MHz–810 MHz). The Virtex-7 FPGA had a total of 433,200 LUTs, 866,400 Flip-Flops, and 3600 DSPs, as well as 52,920 Kb BRAMs.

To efficiently implement the multidimensional reconciliation algorithm, the relationship between hardware resources and throughput needs to be carefully investigated. In view of the available storage resources and editable logical resources, we adopted two approaches in different data processing parts, including the combination of data parallel and time division multiplexing, and the equal-interval circulatory structure-based pipeline operation. These methods can realize the exchange between area and speed. Because the encoding algorithm contains a large number of eight-dimensional matrix operations and the data are 32-bit single-precision floating-point numbers, this consumes a lot of editable logical resources. To overcome these issues, some modules were designed to be implemented with time division multiplexing and the circulatory structure with the same time interval. Due to the storage mode of the FPGA being a one-dimensional array, we converted the matrix form to fit the storage of the FPGA.

**Figure 2:**
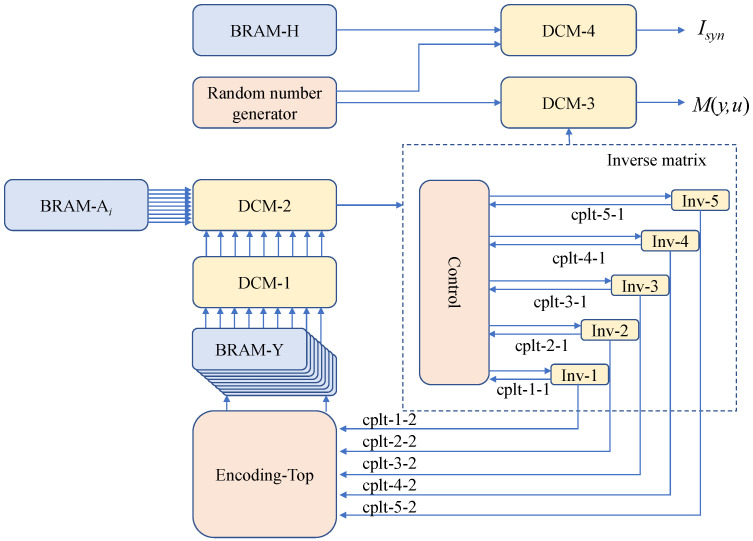
FPGA design for multidimensional reconciliation encoding. The *Control* module switches between different matrix inversion modules according to signal *cplt*-*j*-1 (j∈{1,2,…,5}) and sends a startup flag to module *Inv-j*. Module *Encoding-Top* starts processing new data according to signal *cplt*-*j*-2.

The logic design structure diagram of the multidimensional reconciliation encoding algorithm is shown in Figure 2. *Encoding-Top* is the top-level module of the encoding algorithm. *BRAM-Y* are eight block memory units that are configured as a true dual-port RAM with a width of 32 and depth of 8. *BRAM-A_i_* is a block memory unit that is configured as a true dual-port RAM with a width of 64 and depth of 8 and is used to store eight orthogonal matrices $\left(A_{1},A_{2},\ldots ,A_{8}\right)$. *BRAM-H* is a block memory unit that is configured as a single-port RAM with a width of 18 and depth of 500,000 and is employed to store the check matrix. *DCM* are four different data computing modules. Each *cplt* signal marks the end of the corresponding module operation. *Inv* are five matrix inversion modules with the circulatory structure operating in the same time interval.

#### 3.1.1. Analysis of Data Throughput

The total throughput, $T_{h}$, is a key parameter to evaluate multidimensional reconciliation encoding and can be expressed as

(12) $$\begin{array}{c} T_{h}=\frac{d}{\frac{1}{f_{s}}T_{n}}, \end{array}$$

where $f_{s}$ is the system clock frequency, $T_{n}$ is the number of clock cycles required by the longest pipeline operation step, and *d* is the dimension of multidimensional reconciliation. It can be seen that the total throughput is closely related to the above three parameters. At a given clock frequency, the encoding performance is optimal when *d* = 8, so the throughput is inversely proportional to $T_{n}$. Therefore, with limited hardware resources, the pipeline task block loop should be optimized to achieve high throughput $T_{h}$.

The pipeline operation divides the combinatorial logic into a series of task modules and requires the addition of a first-level register before and after each task module. If the task module is too small, it consumes a large number of registers; each level of registers requires one clock cycle, so it also causes the operation time of the task module to increase. Therefore, we should reasonably divide the pipeline structure according to the specific throughput requirements.

#### 3.1.2. Flexible Division of Pipeline Operation

The goal of the algorithm optimization is to achieve high throughput with minimal FPGA resources. As mentioned above, given the clock frequency and *d* = 8, the number of clock cycles, $T_{n}$, that the longest pipeline step requires is the key factor that affects throughput. We mainly used pipeline technology and a series–parallel structure to balance throughput and resource utilization of the FPGA. Pipeline technology cannot shorten the processing time of a single datum, but it can effectively shorten the processing time of the overall data. In contrast, the parallel structure can shorten the processing time of a single datum.

**Figure 3:**
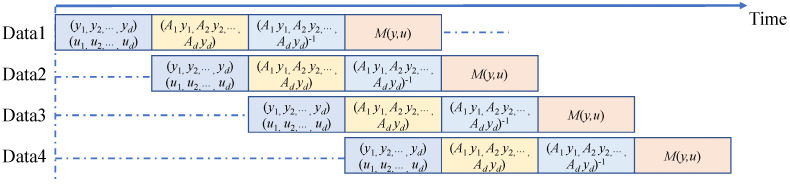
The pipeline structure of the encoding algorithm. The first step is to calculate $\left(y_{1},y_{2},\ldots ,y_{d}\right)$ and $\left(u_{1},u_{2},\ldots ,u_{d}\right)$ using *Y* and $\left(b_{1},b_{2},\ldots ,b_{d}\right)$. The second step is to build standard orthonormal basis $\left(A_{1}y_{1},A_{2}y_{2},\ldots ,A_{d}y_{d}\right)$ for the multidimensional space. The third step is matrix inversion. The fourth step is to map *u* to standard orthonormal basis $\left(A_{1}y_{1},A_{2}y_{2},\ldots ,A_{d}y_{d}\right)$ and obtain M(y,u).

After carefully analyzing the calculation process of the encoding algorithm, we divided the pipeline operation of the encoding operation into four operation steps. Pipelining was used in the normalization of encoding and random number mapping operations, as well as in the syndrome generation module. The module with the longest running time is the calculation of ${\left(A_{1}y_{1},A_{2}y_{2},\ldots ,A_{d}y_{d}\right)}^{-1}$. Figure 3 shows the designed pipeline structure, in which *Data*2 starts to run the first step when *Data*1 has finished the first step. The circulatory structure with the same time interval is employed for the module that computes ${\left(A_{1}y_{1},A_{2}y_{2},\ldots ,A_{d}y_{d}\right)}^{-1}$ to suppress $T_{n}$ according to the required throughput.

### 3.2. Eight-Dimensional Matrix Operation

Because the performance of the multidimensional reconciliation algorithm is optimal when *d* = 8, we only studied the eight-dimensional matrix operation. During the operation process of the eight-dimensional matrix, such as addition, subtraction, multiplication, division, root, square, and inversion [31], the bit growth of data may occur. Although not every level of operation encounters bit growth, each bit growth instance leads to the doubling of the maximum value of the data. This may cause data overflow if fixed-point numbers are employed, making the results of the operation incorrect because the data are not accurate enough. In contrast, floating-point arithmetic has a large dynamic range, and because of its automatic scaling, the possibility of data overflow and mantissa loss can be eliminated. We used 32-bit single-precision floating-point numbers for the calculation of all basic units.

#### 3.2.1. Storage of 8-Dimensional Matrices

According to the addressing mode of the FPGA, the 8×8 matrix is extended to a one-dimensional array of length 64. In order to read and store quickly, we need to use 8 memory units, each with a depth of 8 and a width of 32. The first address of each memory unit stores the first row of the 8-dimensional matrix, and so on. Therefore, each write-and-read operation only takes 8 clock cycles.

#### 3.2.2. Construction of Orthogonal Basis Coordinates

The orthogonal basis coordinates of *u* is $\left(A_{1}y,A_{2}y,\ldots ,A_{d}y\right)$, where $\left(A_{1},A_{2},\ldots ,A_{d}\right)$ is a set of orthogonal matrices that can be computed by four 2×2 matrices [17]. The calculation process is reported below.

The four basis matrices are given by

$$\begin{array}{c} K_{0}=\left(\begin{array}{cc} 1 & 0\\ 0 & 1 \end{array}\right),\;K_{1}=\left(\begin{array}{cc} 0 & 1\\ 1 & 0 \end{array}\right),\\ \;K_{2}=\left(\begin{array}{cc} 0 & -1\\ 1 & 0 \end{array}\right),\;K_{3}=\left(\begin{array}{cc} 1 & 0\\ 0 & -1 \end{array}\right), \end{array}$$

and the orthogonal matrices can be calculated as

(13) $$\begin{array}{c} \left(A_{1},A_{2},\ldots ,A_{8}\right)=\left\{K_{000},K_{332},K_{320},K_{312},K_{200},K_{102},K_{123},K_{121}\right\}, \end{array}$$

where $K_{i_{1},\ldots ,i_{l}}=K_{i_{1}}⊗\cdots ⊗K_{i_{l}}$ represents the tensor product.

According to the characteristic of orthogonal matrix $A_{i}$, each row of it has only one non-zero element, and the absolute value of this element is 1. Therefore, the multiplication of a one-dimensional vector y with a matrix $A_{i}$ can be converted to finding a non-zero element of an orthogonal matrix $A_{i}$ and rearranging the *y* vector. In this way, we can eliminate many multiplication and addition operations and save a lot of LUT resources.

For example,

$$A_{3}=\left(\begin{array}{cccccccc} 0 & 0 & -1 & 0 & 0 & 0 & 0 & 0\\ 0 & 0 & 0 & -1 & 0 & 0 & 0 & 0\\ 1 & 0 & 0 & 0 & 0 & 0 & 0 & 0\\ 0 & 1 & 0 & 0 & 0 & 0 & 0 & 0\\ 0 & 0 & 0 & 0 & 0 & 0 & 1 & 0\\ 0 & 0 & 0 & 0 & 0 & 0 & 0 & 1\\ 0 & 0 & 0 & 0 & -1 & 0 & 0 & 0\\ 0 & 0 & 0 & 0 & 0 & -1 & 0 & 0 \end{array}\right),$$

$$A_{3}\times y=\left(\begin{array}{c} y_{1}\\ y_{2}\\ y_{3}\\ y_{4}\\ y_{5}\\ y_{6}\\ y_{7}\\ y_{8} \end{array}\right)\times \left(\begin{array}{cccccccc} 0 & 0 & -1 & 0 & 0 & 0 & 0 & 0\\ 0 & 0 & 0 & -1 & 0 & 0 & 0 & 0\\ 1 & 0 & 0 & 0 & 0 & 0 & 0 & 0\\ 0 & 1 & 0 & 0 & 0 & 0 & 0 & 0\\ 0 & 0 & 0 & 0 & 0 & 0 & 1 & 0\\ 0 & 0 & 0 & 0 & 0 & 0 & 0 & 1\\ 0 & 0 & 0 & 0 & -1 & 0 & 0 & 0\\ 0 & 0 & 0 & 0 & 0 & -1 & 0 & 0 \end{array}\right)=\left(\begin{array}{c} -y_{3}\\ -y_{4}\\ y_{1}\\ y_{2}\\ y_{7}\\ y_{8}\\ -y_{5}\\ -y_{6} \end{array}\right).$$

#### 3.2.3. Inverse Matrix of the Eight-Dimensional Matrix

Because $\left(A_{1},A_{2},\ldots ,A_{d}\right)$ is a group of eight orthogonal matrices, $\left(A_{1}y_{1},A_{2}y_{2},\ldots ,A_{d}y_{d}\right)$ is invertible. The calculation of M(y,u) can be written as

(14) $$\begin{array}{c} M\left(y,u\right)=\sum_{i=0}^{d}\alpha_{i}\left(y,u\right)A_{i}={\left(A_{1}y_{1},A_{1}y_{1},\ldots ,A_{d}y_{d}\right)}^{-1}u. \end{array}$$

**Figure 4:**
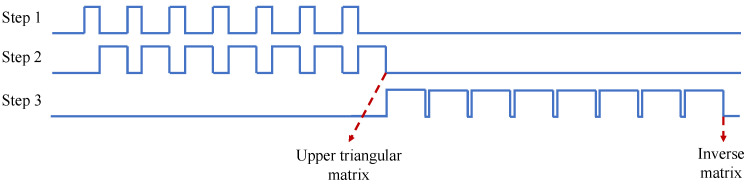
Time division multiplexing of inverse matrix. *Step* 1 is finding the maximum value of each row. *Step* 2 is elementary row operations. *Step* 3 is data normalization.

For the calculation of ${\left(A_{1}y_{1},A_{2}y_{2},\ldots ,A_{d}y_{d}\right)}^{-1}$, we used the Gaussian elimination method [32] to obtain the inverse matrix of the eight-dimensional matrix, which can be divided into three steps: (1) finding the maximum value of each row; (2) obtaining an upper triangular matrix using elementary row operations; (3) data normalization. Due to the calculation of the inverse matrix consuming a lot of LUTs and DSPs, we used time division multiplexing to balance the speed and area of the FPGA, as shown in Figure 4. *Step* 1 and *Step* 2 perform loop execution seven times to obtain an upper triangular matrix. Then, *Step* 3 performs loop execution eight times to obtain an inverse matrix. *Step* 1 sends the position of the maximum value for each row to *Step* 2, and *Step* 2 sends the matrix to *Step* 1 after elementary row operations.

In order to search for the maximum value of each row, eight comparators and an AND door are exploited. During the calculation process, the eight data in each row are simultaneously read from the same address. Because the comparator consumes only 87 LUTs, seven comparators are run concurrently and then the AND gate is used to decide the maximum value in each row. In this way, the logic delay of this combination circuit is minimized, which consists of the delay of the comparator of single-precision floating-point numbers and the delay of an AND gate.

#### 3.2.4. Multiplexing Structure of Matrix Inversion Module

**Figure 5:**
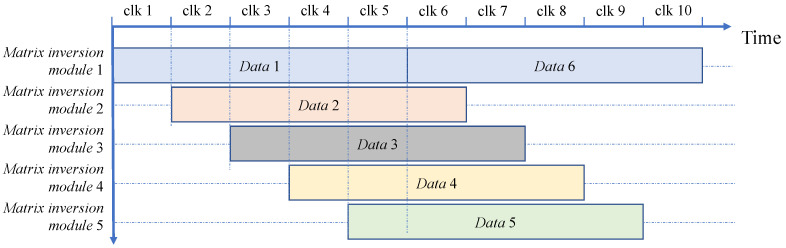
Circulatory structure with the same time interval. *clk* has the clock cycle of $T_{n}$. Each *Matrix inversion module j* is multiplexed.

The matrix inversion module cannot be subdivided into multi-level pipelines with the pipeline operation due to module multiplexing and nested loops. To improve the computation speed, we designed a circulatory structure with the same time interval, as shown in Figure 5.

Firstly, $T_{n}$ is calculated based on the target throughput. The input data control module then assigns the input data stream to different matrix inversion modules simultaneously. Then, *Data* 2 performs the matrix inversion operation after $T_{n}$ clock cycles relative to *Data* 1, and so on. Therefore, *Data* 1 and *Data* 6 are both calculated by *Matrix inversion module* 1. Each data stream has a corresponding matrix inversion module with a time interval of $T_{n}$. The number of reused matrix inversion modules is $\frac{T_{inv}}{T{}_{n}}$, where $T_{inv}$ is the time required for the inversion of an eight-dimensional matrix in serial operation.

### 3.3. Construction of the Syndrome

Due to the existence of channel loss and excess noise in quantum key distribution, there is inevitably some discrepancy when the two groups of Gaussian numbers of Alice and Bob are transformed into binary bits in the spherical space. To correct the bit errors, Bob can send a syndrome of his bit string to Alice. Here, the syndrome can be obtained by multiplying the check matrix with Bob’s random bit string. The check matrix of an LDPC code [22] has very strong sparsity, that is, the “0” element accounts for most of the check matrix and the “1” element is very sparsely distributed. Considering the sparsity characteristic of an LDPC code, the shifting method is used to calculate the syndrome.

The storage of the entire check matrix requires a large amount of resources. In order to save memory resources, we store the check matrix by storing the positions of non-zero elements in each row of the matrix. The check matrix is extended in a quasi-cyclic manner [33]. Notice that the shifting operation is implemented after quasi-cyclic expansion, in which the non-zero element is extended to a 16 × 16 identity matrix. So, we use an 18-bit-wide memory unit to save the basis matrix and the extended matrix. The first 14 bits ($A_{0}$–$A_{13}$) describe the positions of non-zero elements in the basis matrix, and the last four bits ($S_{0}$–$S_{3}$) describe the shifting of the extended matrix.

**Figure 6:**
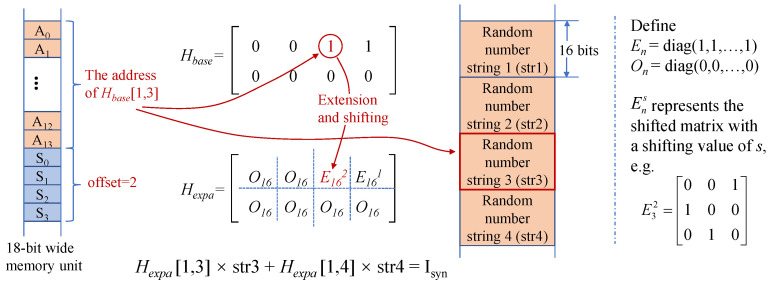
Calculation procedure of the syndrome for a 2×4 base matrix. $H_{base}$ and $H_{expa}$ denote the base matrix and the matrix extended in a quasi-cyclic manner. str1, str2, str3, and str4 denote independent random bit strings.

For the multiplication of the basis matrix and the input random bit string, the position of the non-zero elements of the basis matrix is used as the address to read the bit string, and the drifting value of the non-zero element is used to shift the bit string. The multiple shifted bit strings are accumulated to obtain the product of a row of the check matrix and the random bit string, that is, the syndrome, as shown in Figure 6. If the address of the Nth non-zero element is smaller than the address of the (N−1)th non-zero element, that means that we start to compute the next syndrome, which is the product of the next row of the check matrix and the random bit string. This saves a lot of DSPs and LUTs and increases the running rate, because a lot of multiplication and addition operations are omitted.

## 4. Results and Discussions

**Figure 7:**
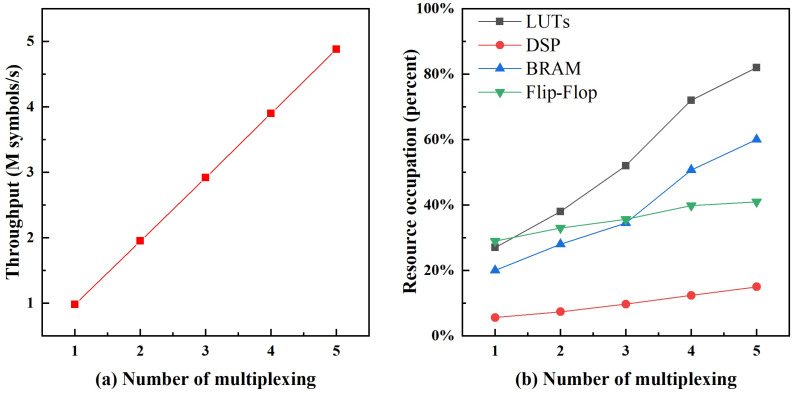
Resource consumption and data throughput for different multiplexing structures.

In this section, we show the implementation results of multidimensional reconciliation encoding on an FPGA chip (Xilinx Virtex-7 FPGA). The feasibility of the encoding algorithm was validated. When the pipeline step takes longer than $T_{n}$, we can use a modified ping-pong operation to multiplex the step. In this case, throughput $T{}_{h}$ (as shown in (11)) becomes

(15) $$\begin{array}{c} T_{h}=\frac{nd}{\frac{1}{f_{s}}T_{n}}, \end{array}$$

where *n* is the number for multiplexing. According to all the relevant parameters [15], the practical secret key rate of a CV-QKD system is given by

(16) $$\begin{array}{c} K_{prac}=\alpha \left(1-FER\right)\left(\frac{n}{N}\right)\left(\beta I_{AB}-\chi_{BE}-\Delta \left(n\right)\right), \end{array}$$

where $\alpha =PP_{out}/PP_{in}$; $PP_{out}$ and $PP_{in}$ represent the post-processing output and input rates, respectively; FER is the frame error rate; *n* is the number of data used to distill the secret key; *N* is the number of sifted data after quantum transmission and measurement; $I_{AB}$ is the mutual information between Alice and Bob; $\chi_{BE}$ is the Holevo bound; and Δ(n) is the finite-size offset factor. Notice that $PP_{out}$ depends on $T_{h}$ and satisfies $PP_{out}<=T_{h}$; therefore, coding throughput $T_{h}$ affects the secret key rate of the CV-QKD system through α. The consumed resources and throughput for different multiplexing structures are shown in Figure 7.

We can see that the data throughput and hard resources consumed increased with the number of multiplexing. A throughput of 0.98 Msymbols/s was achieved without multiplexing. The throughput increased to 4.88 M symbols/s when the number of multiplexing was five. The resource consumption of LUTs was the highest, whereas the resource consumption of DSPs was the lowest.

The throughput results of the multidimensional reconciliation encoding algorithm on an FPGA and a CPU are shown in Table 1. The model of the CPU was MXC-6301D(EA), running with the C programming language. It is evident that the FPGA-based encoding algorithm dramatically improved the data processing speed in comparison to the CPU-based encoding algorithm, which had a throughput of only 0.063 M per second.

Our proposed algorithm for obtaining the syndrome can be adapted to systems with different parity-check matrices. The simulated results on an FPGA are shown in Table 2. Three check matrices with two different rates (0.02 and 0.1) and two different code lengths (160,000 and 500,000) were used to calculate the syndrome. For a fixed code rate, the time taken was directly proportional to the code length. For a fixed code length, the time taken was slightly longer when the code rate was higher.

## 5. Conclusions

In this paper, we propose and demonstrate an FPGA-based multidimensional reconciliation encoding algorithm with variable data throughput that is suitable for applications in the real-time data post-processing of CVQKD systems. The syndrome construction algorithm consumes very little BRAMs and LUTs of the FPGA and can adapt to parity-check matrices with different code rates and code lengths. It is noted that the achieved data throughput is still limited. In our current algorithm, we find that matrix inversion consumes lots of hardware resources, which hinders the further improvement of throughput. In the future, we will improve the matrix inversion algorithm to make it compatible with pipeline operations; combined with higher system clock speed of the FPGA, a multidimensional reconciliation encoding algorithm with much higher throughput is possible.

## Figures and Tables

**Table 1:** Comparison of the throughput of the multidimensional reconciliation encoding algorithm between an FPGA and a CPU.

Computing Platform	Throughout (M Symbols/s)
FPGA (Xilinx Virtex-7)	4.88
CPU (MXC-6301D(EA))	0.063

**Table 2:** Time taken for constructing the syndrome using different code rates and code lengths.

Rate	Length	Time (s)
0.1	160,000	0.00452
0.1	500,000	0.00904
0.02	500,000	0.00801

## Data Availability

Not applicable.
